# Expression profiles and potential roles of transfer RNA‐derived small RNAs in atherosclerosis

**DOI:** 10.1111/jcmm.16719

**Published:** 2021-06-16

**Authors:** Xiangqin He, Yanyan Yang, Qi Wang, Jueru Wang, Shifang Li, Chunrong Li, Tingyu Zong, Xiaolu Li, Ying Zhang, Yulin Zou, Tao Yu

**Affiliations:** ^1^ Department of Cardiac Ultrasound The Affiliated Hospital of Qingdao University Qingdao China; ^2^ Department of Immunology Basic Medicine School Qingdao University Qingdao China; ^3^ Department of Cardiology The First Affiliated Hospital of Xi’an Jiaotong University Xi'an, Shaanxi China; ^4^ The department of thyroid surgery The Affiliated Hospital of Qingdao University Qingdao China; ^5^ Department of Neurosurgery The Affiliated Hospital of Qingdao University Qingdao China; ^6^ Institute for Translational Medicine The Affiliated Hospital of Qingdao University Qingdao China

**Keywords:** atherosclerosis, cell proliferation, expression profile, non‐coding RNAs, tRNA‐derived fragments

## Abstract

Knowledge regarding the relationship between the molecular mechanisms underlying atherosclerosis (AS) and transfer RNA‐derived small RNAs (tsRNAs) is limited. This study illustrated the expression profile of tsRNAs, thus exploring its roles in AS pathogenesis. Small RNA sequencing was performed with four atherosclerotic arterial and four healthy subject samples. Using bioinformatics, the protein‐protein interaction network and cellular experiments were constructed to predict the enriched signalling pathways and regulatory roles of tsRNAs in AS. Of the total 315 tsRNAs identified to be dysregulated in the AS group, 131 and 184 were up‐regulated and down‐regulated, respectively. Interestingly, the pathway of the differentiated expression of tsRNAs in cell adhesion molecules (CAMs) was implicated to be closely associated with AS. Particularly, tRF‐Gly‐GCC might participate in AS pathogenesis via regulating cell adhesion, proliferation, migration and phenotypic transformation in HUVECs and VSMCs. In conclusion, tsRNAs might help understand the molecular mechanisms of AS better. tRF‐Gly‐GCC may be a promising target for suppressing abnormal vessels functions, suggesting a novel strategy for preventing the progression of atherosclerosis.

## 1 INTRODUCTION

Atherosclerosis (AS), one of the most common cardiovascular diseases, is the primary cause of morbidity and mortality in the case of cardiac events, thus posing a great threat to human health worldwide.^1^, ^2^, ^3^ The disease is characterized by deposition of lipid and other blood components in the intima of the artery, proliferation of smooth muscle cells and increase of collagen fibres, chronic inflammation, plaque formation and hardening of the vascular walls.^3^, ^4^, ^5^ The development of atheromatous plaques leads to serious clinical outcomes.^6^, ^7^ Once the vulnerable plaques rupture, they induce several clinical conditions, including stroke and myocardial infarction.^8^ To date, arteriography is considered to be the gold standard for the diagnosis of AS. However, given the involvement of an invasive procedure, primary injury is mostly unavoidable.^9^ Since the majority of the available evidence focuses on the pathogenesis of AS,^10^, ^11^, ^12^ the specific molecular mechanism underlying the pathogenesis of AS remains unclear. In order to ensure early diagnosis and improve clinical outcomes and provide new insights into targeted therapies, identification of the key molecules involved in the pathogenesis of AS is warranted.

Transfer RNAs (tRNAs) play a crucial role in protein synthesis by transporting activated amino acids to the ribosome.^13^ A heterogeneous population of small non‐coding RNAs (ncRNAs) with lengths of 18‐40 nucleotides cleaved from tRNA was found^14^ and named as tRNA‐derived small RNAs (tsRNAs). It is derived from cleavage of mature and precursor tRNAs at different cleavage sites and generally classified into two groups: tRNA‐related fragments (tRFs) and tRNA halves (tiRNAs).^15^ Depending on the length of tRFs, they are normally grouped into four subclasses as follows: tRF‐1, tRF‐2, tRF‐3 and tRF‐5, and two novel categories of tRFs such as i‐tRFs^16^ and 5’ leader‐exon tRFs^17^ were reported recently. Meanwhile, based on the specific lengths, tRF‐5 is further classified as follows: tRF‐5a, tRF‐5b and tRF‐5c. The tRF‐3 is divided into tRF‐3a and tRF‐3b. Mature tRNA digested by angiogenin, Dicer or other RNases yield tRF‐2, tRF‐3, tRF‐5 and tiRNAs, while RNase Z produces tRF‐1.^18^, ^19^ Recent studies revealed that tsRNAs are localized in cell lines, tissues or extracellular body fluids, exerting biological functions through a variety of mechanisms.^20^ The tsRNAs can interact with proteins or messenger RNA (mRNA), leading to regulation of gene expression and chromatin and epigenetic modifications. Conventionally, they remain associated with cell cycle, cell proliferation, cell apoptosis, RNA stability and degradation, and DNA damage response.^21^, ^22^ Recent studies demonstrated that ncRNAs including tsRNAs contribute to the pathology of various diseases, including tumour suppression, infectious diseases, metabolic disorder and neurodegeneration.^23^, ^24^, ^25^ However, the potential role and associated mechanisms of tsRNAs in AS are yet to be unravelled.

In this study, the expression profile of tsRNAs in AS tissue samples was assessed using RNA sequencing technologies. Thereafter, tsRNAs/mRNA interaction networks were constructed using bioinformatics, and the potential role of the target genes of candidate tsRNAs in the pathogenesis of AS was predicted. Results showed that cell adhesion molecules could be main targets involved in differentially expressed tsRNAs. Particularly, tRF‐Gly‐GCC exhibited the promising capacity in regulating biological functions of human umbilical vein endothelial cells (HUVECs) and vascular smooth muscle cells (VSMCs), which might participate in AS pathogenesis. Better understanding of the systematic regulatory networks in AS could offer novel and potential diagnostic markers and therapeutic targets for timely and accurate detection and treatment of AS.

## 2 MATERIALS AND METHODS

### 2.1 Sample collection

A total of 14 atherosclerotic arterial samples and 16 healthy samples were collected from Affiliated Hospital of Qingdao University in 2020. The atherosclerotic arterial tissues were obtained from patients diagnosed with AS and ones who underwent carotid endarterectomy. Arteries from healthy subjects who died in traffic accident were also collected. The obtained tissues were frozen in liquid nitrogen until total RNA was extracted. The study was approved by the Ethics Committee of the Affiliated Hospital of Qingdao University.

### 2.2 Cell culture

HUVECs and VSMCs were purchased from the Chinese Type Culture Collection (Shanghai, China). HUVECs were cultured in Dulbecco's modified Eagle medium (DMEM)/F‐12 (GIBCO) supplemented with 10% foetal bovine serum (FBS; ExCell Bio), and DMEM with 10% FBS was supplemented for VSMCs. The cells were cultured with 5% carbon dioxide (CO_2_) at 37°C and were trypsinized to generate single cell suspensions at 80% confluency.

### 2.3 Total RNA extraction

Total RNA collected from human healthy (from 12 individuals) and atherosclerotic arteries (from 10 patients) were extracted using TRIzol Reagent (Sparkjade Science Co., Ltd.,), according to the manufacturers’ guidelines. The quantity and concentration of RNA samples were checked using NanoDrop ND‐1000 instrument (Thermo Fisher Scientific). Integrity was assessed by agarose gel electrophoresis.

### 2.4 RNA sequencing

Four atherosclerotic arterial and four healthy subject samples were used for RNA sequencing. Before library preparation, each of the RNA samples was subjected to the following treatments: 3’‐aminoacyl (charged) deacylation to 3’‐OH for 3’‐adaptor ligation, 3’‐cP (2’,3’‐cyclic phosphate) removal to 3’‐OH for 3’‐adaptor ligation, 5’‐OH (hydroxyl group) phosphorylation to 5’‐P for 5’‐adaptor ligation, and m1A and m3C demethylation for efficient reverse transcription. These treatments prevented RNA modifications that interfere with the construction of small RNA sequencing library. Subsequently, the sequencing libraries were size‐selected using an automated gel cutter for the RNA biotypes to be sequenced. Thereafter, 3’‐adapter and 5’‐adapter ligation, complimentary DNA (cDNA) synthesis and library PCR amplification were performed. The prepared sequencing library was absolutely quantified using the Agilent Bioanalyzer 2100 (Invitrogen). Illumina NextSeq 500 (Illumina) was used for sequencing; sequencing type was 50‐bp single‐read at 10 M reads.

### 2.5 Data analysis

Illumina chastity filter was used to obtain the raw sequencing data quality control. The 5’‐ and 3’‐adaptor bases of the sequencing reads were trimmed using the Cutadapt software to filter over 15 nucleotides. NovoAlign software (v2.07.11) was used to arrange the trimmed reads of the mature‐tRNA sequences, while BowTie software aligned the unmapped reads to the pre‐tRNA sequences (Langmead et al, 2009). Expression level of the tsRNAs was normalized as counts per million (CPMs). Based on R package, the expression profile with average CPM was identified. Moreover, the differentially expressed tsRNAs between the control and AS groups were screened out based on the following criteria: fold change (FC)>1.5 and *P*‐value < .05. Hierarchical clustering, scatter plots, pie plots, Venn plots and volcano plots were constructed for the tsRNAs using R or Python to obtain a visual representation.

### 2.6 Bioinformatic analysis

In order to understand the underlying mechanisms and functions of the candidate tsRNAs, it is crucial to investigate the target genes of tsRNAs. TargetScan and miRanda were applied to predict the target genes of the candidate tsRNAs. The targets were obtained based on the overlapping results from the two websites. Further, Cytoscape software was used to visualize the corresponding interactions. The Database for Annotation, Visualization and Integrated Discovery (DAVID) was performed to achieve gene ontology (GO) annotation enrichment analyses and to exhibit the biological processes (BPs), cellular components (CCs) and molecular functions (MFs) of the target mRNAs. Kyoto Encyclopedia of Genes and Genomes (KEGG) pathway enrichment analyses revealed the significant pathways in which the target genes were enriched.

### 2.7 Protein‐protein interaction (PPI) network construction

The STRING tool was used to visualize the PPI network and to analyse and demonstrate the molecular mechanisms of the related targets. In the PPI network, nodes represent the target genes and lines between the nodes are indicative of the strength of the associated interactions between the two nodes. Colour of the lines represents the strength of interaction. The hub genes were defined as genes which exert important influence on the network. They were distinguished based on the criteria of interaction degree, calculated by CytoHubba in Cytoscape (http://cytoscape.org/). The corresponding interactions were visualized using Cytoscape software.

### 2.8 RT‐qPCR analysis

The tsRNAs chosen for validation by RT‐qPCR were selected based on the following criteria: tsRNAs with exact match of alignment information, FC>1.5, *P*‐value < .05 and expression detected in all samples, indicating that the tag count for each sample was over zero. Based on these criteria, six up‐regulated tRFs/tiRNAs of tRF‐1:28‐Gly‐GCC‐4, tRF‐1: tRF‐1:31‐Glu‐TTC‐2, tRF‐1:16‐Gly‐GCC‐1, tRF‐+1:T23‐Arg‐TCG‐1, tRF‐53:71‐chrM. Gly‐TCC and tRF‐51:71‐chrM were chosen for RT‐qPCR. The cDNA was synthesized using the reverse transcription kit (Takara), and SYBR Green PCR Master Mix (Yeasen Biotechnology) was selected for RT‐qPCR accomplishment. The tRNA expression was normalized with U6 (Takara). Fold changes in tsRNAs expression were calculated using the 2^−ΔΔCt^ method for each sample in triplicate. ΔΔCt = [(Ct tRNA‐Ct U6) diseased ‐ (Ct tRNA‐Ct U6) control]. The primers used to amplify the tRF transcripts are presented in Table S1.

### 2.9 Cell adhesion of monocytes to HUVECs

HUVECs (2 × 10^4^ cells) were seeded and incubated in 48‐well plates and treated with 20 μmol/L RNAs for 24 hours. Then, THP‐1 cells (1 × 10^6^ cells) were stained with 1 μmol/L carbox fluorescenceindiacetate succinimidyl ester (CFSE) (MedChem Express) in 0.1% BSA for 10 minutes at 37°C and ended with PBS for 10 minutes. And the cells were plated in HUVECs for a 4 hours co‐incubation at 37°C. Non‐adherent cells were removed by washing with PBS for three times; adhered THP‐1 cells were visualized by Fluorescence microscope (olympusIX73, Japan) and counted from 3 randomly selected fields.

### 2.10 Cell counting Kit‐8 assay

Proliferation ability of HUVECs and VSMCs was evaluated using the Cell Counting Kit‐8 (CCK8; 7sea Biotech). HUVECs and VSMCs were transfected for 24 hours in 96‐well plates. Thereafter, 10 μL of CCK8 solution was added to each well and incubated for 1 hours at 37°C. Finally, the absorbance at 450 nm, reflecting the cell proliferative potential, was measured and analysed. Each experiment was repeated three times.

### 2.11 Transwell assay

HUVECs and VSMCs were transfected in 6‐well plates for 24 hours. Thereafter, the cells were treated with serum‐free DMEM for 12 hours and incubated in the upper chamber of transwell insert (Corning), which was again treated with 200 µL of serum‐free medium and 500 µL of DMEM supplemented with 10% FBS in the lower chamber, with 5% CO_2_ at 37°C for 24 hours. Finally, the cells were fixed with 4% paraformaldehyde for 1 hour and stained with 0.1% crystal violet for 30 minutes. The cells that migrated were photographed using a microscope (Nikon Ti‐s). Each experiment was repeated three times.

### 2.12 Wound healing assay

The tRF‐Gly‐GCC mimics, tRF‐Gly‐GCC inhibitor and normal control (NC) were transfected for 24 hours when VSMCs were cultivated to 80% density in 6‐well plates. A 1000 μL pipette tip was stroked through the centre of the well to make a wound. The cells were then washed twice using PBS and treated with 2% FBS supplemented DMEM. In order to calculate the migration rate, distance of the wound at 0, 12, 24 and 36 hours compared to that at 0 hour was imaged. Each experiment was repeated three times.

### 2.13 Western blot

Western blot assay was performed, as described previously but with minor modifications.^26^, ^27^ Briefly, VSMCs were lysed in RIPA buffer (Epizyme) containing 10% phenylmethylsulfonyl fluoride and 1% protease inhibitor cocktail. Thereafter, the BCA protein assay kit was used to quantify the total protein quality. The polyvinylidene fluoride membranes with protein transferred after a sodium dodecyl sulphate polyacrylamide gel electrophoresis were incubated with alpha‐smooth muscle actin (α‐SMA), major histocompatibility complex (MHC) and calponin antibodies at 4°C overnight. The membrane was again washed thrice and incubated with corresponding secondary antibody for 1 hour at room temperature. Finally, the antigen‐antibody complexes were visualized with enhanced chemiluminescence.

### 2.14 Statistical analysis

Data were presented as mean ± standard deviation. Student's *t* test was carried out to compare the control and AS groups. One‐way analysis of variance followed by Bonferroni post hoc test was performed for three or more groups. Results were visualized using GraphPad Prism 8. A *P*‐value < .05, *P‐value < .05, **P < .01 and ***P‐value < .001 were considered significant.

## 3 RESULTS

### 3.1 Differential expression profiles of tRF and tiRNA in AS and NC groups

**FIGURE 1:**
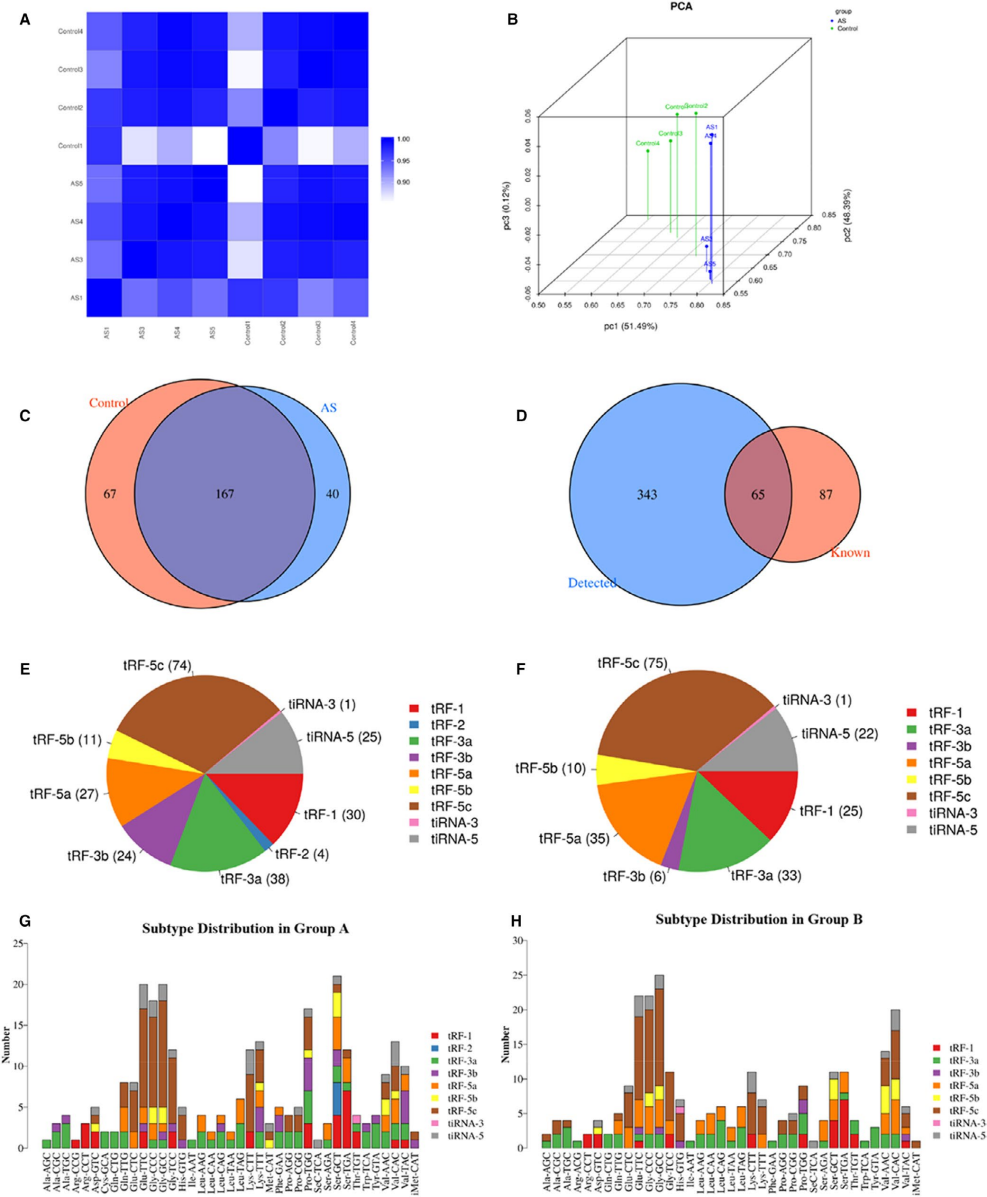
Differential expression profiles of transfer RNA (tRNA)‐derived fragments and tRNA halves in atherosclerosis (AS) and normal control (NC) groups. A, Heatmap of correlation coefficient in AS and NC groups. Colour in the panel represents the correlation coefficient. B, Primary component analysis. The X, Y and Z axes represent the three main factors that influence sample expression. C, Venn diagram of common and specifically expressed tRF and tiRNA in both the groups. D, Venn diagram of tRF and tiRNA known and detected. E, The distribution of tRF and tiRNA subtypes in NC group. F, The distribution of tRF and tiRNA subtypes in AS group. G, The number of subtypes of tRF and tiRNA in normal control group. H, The number of subtypes of tRF and tiRNA in atherosclerosis group. A: normal control, B: atherosclerosis

In order to assess the expression profiles of tRF and tiRNA in control and AS groups, sequencing quality was examined using the quality (Q) score plot of the samples. Samples with a Q score>30 (>99.9% correct) were defined as high‐quality data. Results revealed that more than 90% of the samples had a Q ≥ 30 (Table S2). Then, the correlation coefficient, a significant evaluation criterion, was calculated for any two of all samples based on the expression level. Samples of two groups showed a high degree of similarity (Figure 1A). After analysis of the correlation coefficient, principal component analysis method was used to explore the sample classes that showed different tRF and tiRNA expression profiles in the two groups (Figure 1B).

### 3.2 Types of tRFs and tiRNAs expressed in AS and NC groups

**FIGURE 2:**
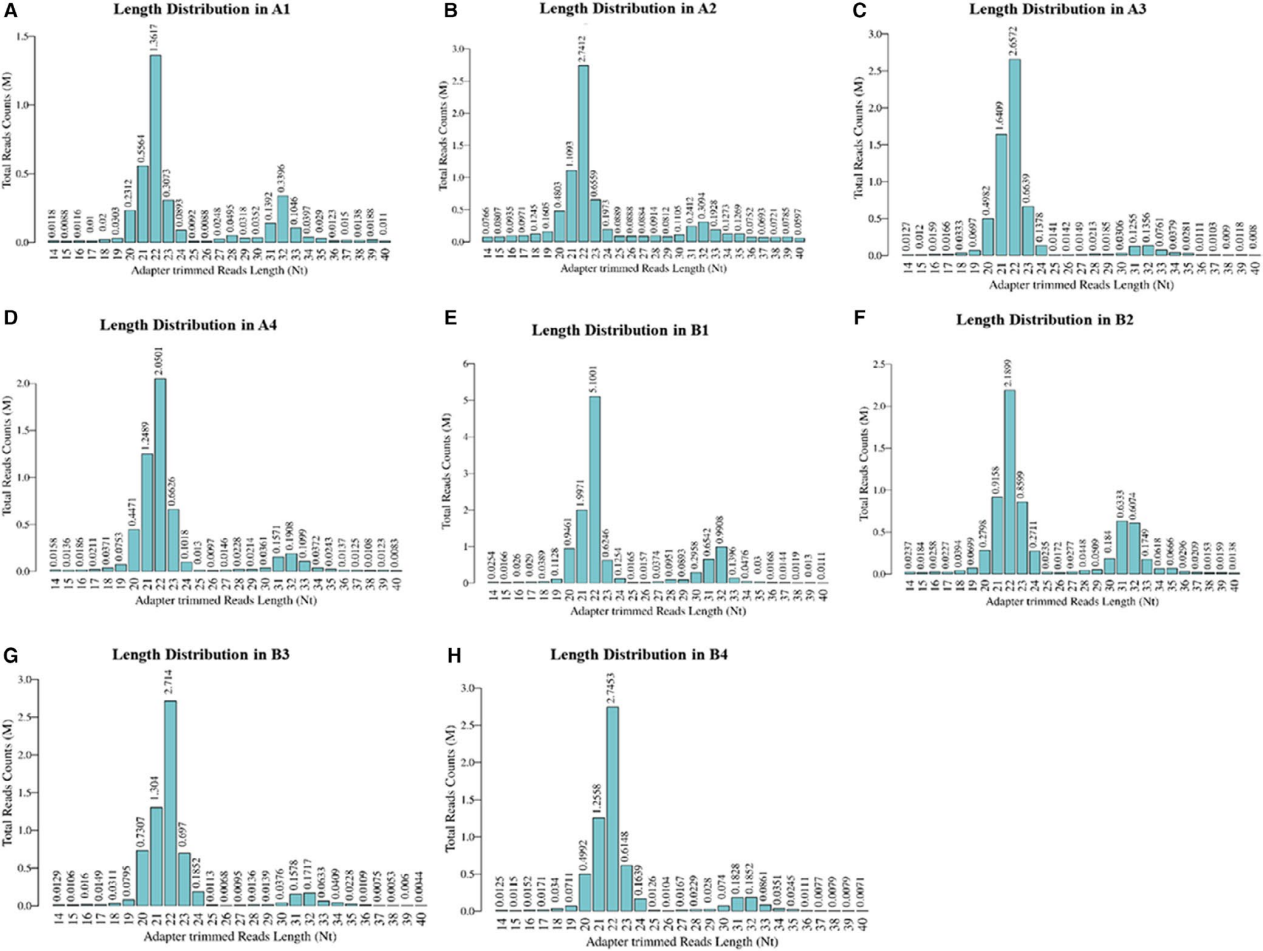
Length of the transfer RNA (tRNA)‐derived fragments (tRF) and tRNA halves (tiRNA) in atherosclerosis (AS) and normal control (NC) groups. Bar chart showing the total read counts against the read lengths in AS and NC groups, respectively. A: normal control, B: atherosclerosis

A total of 408 tRFs and tiRNAs were identified between the AS and NC groups. Venn diagram indicated that 167 tRFs and tiRNAs were commonly expressed in both the groups, 40 tsRNAs were particularly expressed in the AS group, and 67 tsRNAs were expressed only in the NC group (Figure 1C). In particular, compared with tRFdb, a database for tRNA fragments, the Venn diagram, revealed that 65 tRFs and tiRNAs were known (Figure 1D). To investigate the distribution of tRF and tiRNA subtypes in both the groups, a pie chart was prepared. It demonstrated an increase in the expression of tRF‐5a and an obvious decrease in the expression of tRF‐3b in AS group (Figure 1E,F). Moreover, tRF‐2 was uniquely expressed in NC group and not in the AS group. The tRNA isodecoders were observed to possess the same anticodon but different body sequence, leading to tRFs or tiRNAs with identical sequences which might be derived from different tRNA genes based on the tRFs. The numbers of subtype tRF and tiRNA against the tRNA isodecoders are shown by stacked bar chart. The main subtypes of tRF and tiRNA are tRF‐5c and tRF‐3a in NC and AS groups, respectively; tRF‐3b was obviously reduced in the AS group compared to the NC group (Figure 1,H). The lengths of the tRF and tiRNA in AS and NC groups were compared in stacked bar charts. Results demonstrated that the sequence read length mostly varied between 20 and 24 nucleotides (Figure 2).

### 3.3 Differential expression of tRFs and tiRNAs in AS and NC groups

**FIGURE 3:**
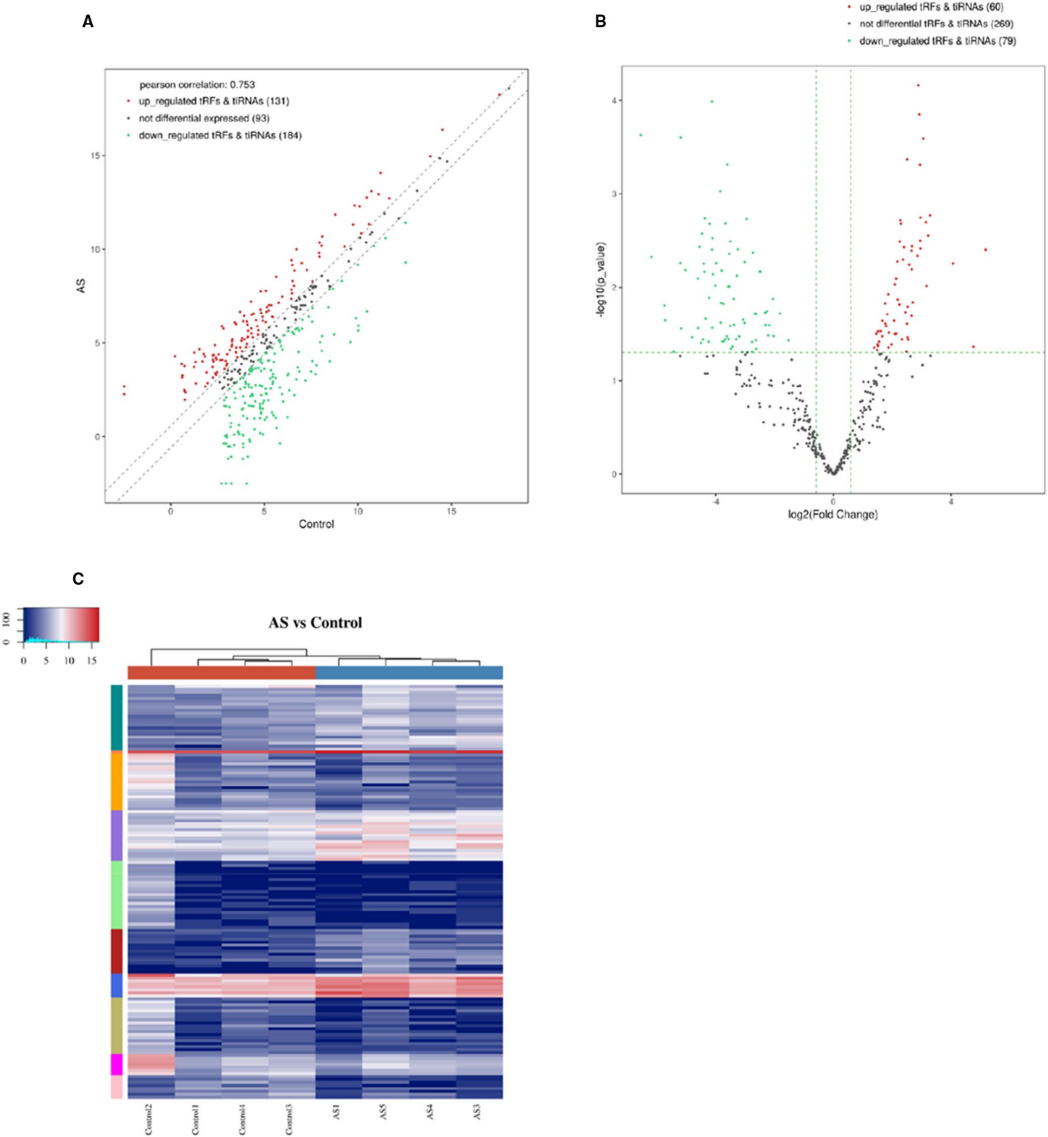
Differential expression of transfer RNA (tRNA)‐derived fragments (tRFs) and tRNA halves (tiRNAs) in atherosclerosis (AS) and normal control (NC) groups. A, Scatter plots for AS and NC groups. Count per million value of all tRFs and tiRNAs. B, Volcano plot for differential expression in AS and NC groups. C, The hierarchical clustering heatmap for tRF and tiRNA between the two groups. Each row represents one tRNA and each column represents one sample

A total of 315 tRFs and tiRNAs were identified to be dysregulated in the AS group. Among them, expressions of 131 tsRNAs were enhanced and 184 were reduced compared to that of the NC group, results are shown by scatter plots in Figure 3A. Thereafter, the tsRNAs were screened (FC>1.5 and *P*‐value < .05); 179 tsRNAs did not meet the criteria and hence were excluded. Among the ones that complied, 60 tsRNAs were up‐regulated and 79 were down‐regulated. Volcano plot was used to visualize the variation in expression (Figure 3B). At the same time, hierarchical clustering toadied in visualization of the tsRNAs with significant differences in the expression levels, following the same criteria. The distinguishable tsRNAs expression profiles in AS and NC groups are shown in Figure 3C.

### 3.4 Validation of the diverse expression levels of tRFs and tiRNAs

**FIGURE 4:**
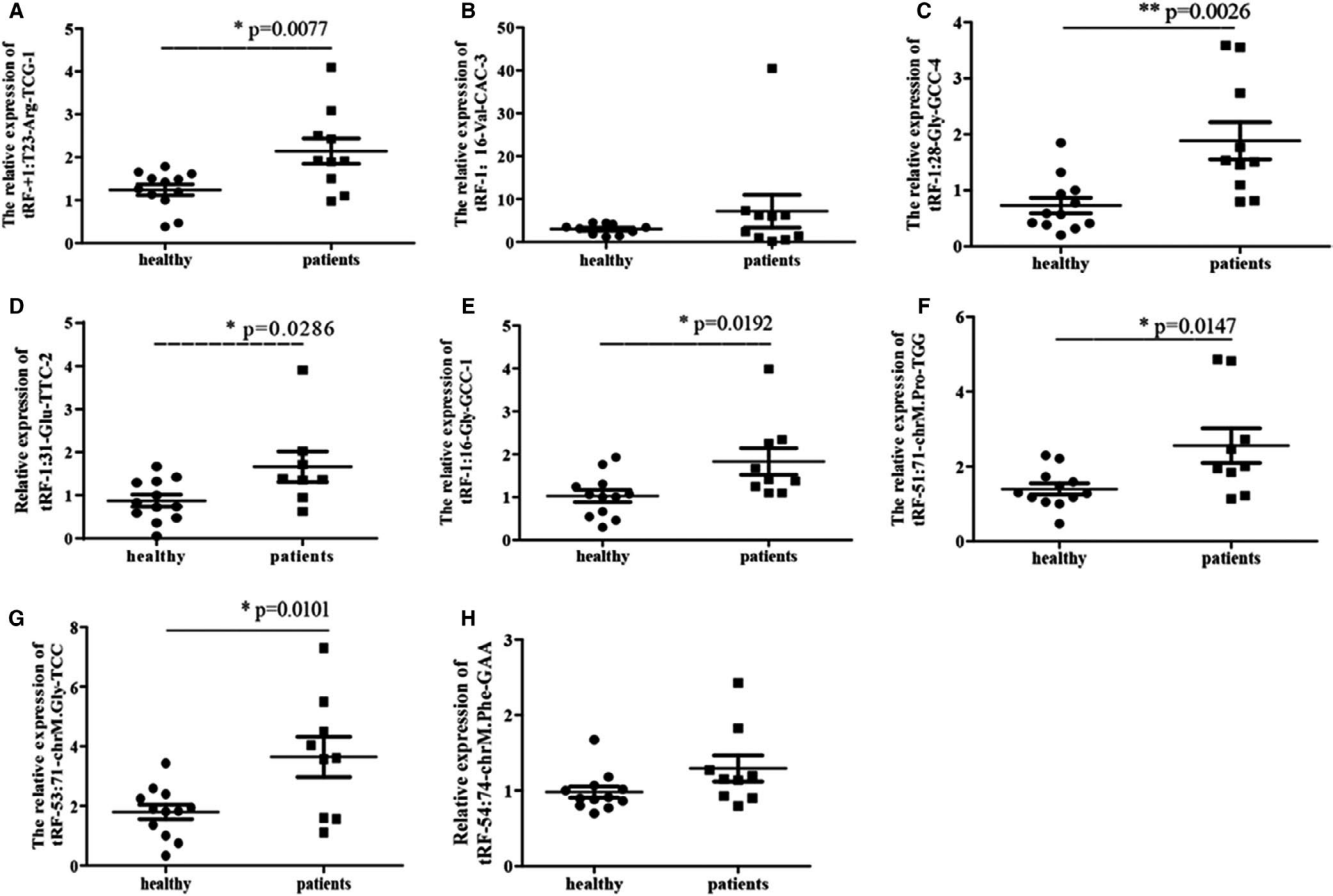
Validation of the diverse expression level of transfer RNA (tRNA)‐derived fragments and tRNA halves in clinical samples. Healthy: normal control group, patients: atherosclerosis group

In order to validate the expression levels of identified tRFs and tiRNAs, the differentially expressed candidates with high *P*‐value in 10 atherosclerotic arterial and 12 healthy subjects were quantified using RT‐qPCR. However, the results showed no significant differences between tRF‐1:16‐Val‐CAC‐3 and tRF‐54:74‐chrM. Phe‐GAA expression between the two groups and were hence excluded from further analysis. Furthermore, RT‐qPCR results indicated that tRF‐1:28‐Gly‐GCC‐4, tRF‐1:31‐Glu‐TTC‐2, tRF‐1:16‐Gly‐GCC‐1, tRF‐+1:T23‐Arg‐TCG‐1, tRF‐53:71‐chrM. Gly‐TCC and tRF‐51:71‐chrM. Pro‐TGG were significantly up‐regulated in the AS group (Figure 4), whereas there was no obvious effect on tRNAs (Figure S1). Based on these data, tRF‐1:28‐Gly‐GCC‐4 (tRF‐Gly‐GCC) was selected for the further cellular experiments. Expression of tRF‐Gly‐GCC in AS group was twice that of the control group (*P*‐value = .0026) and it was identified as the potential regulator of tRFs and tiRNAs.

### 3.5 tRF‐target gene interactions

**FIGURE 5:**
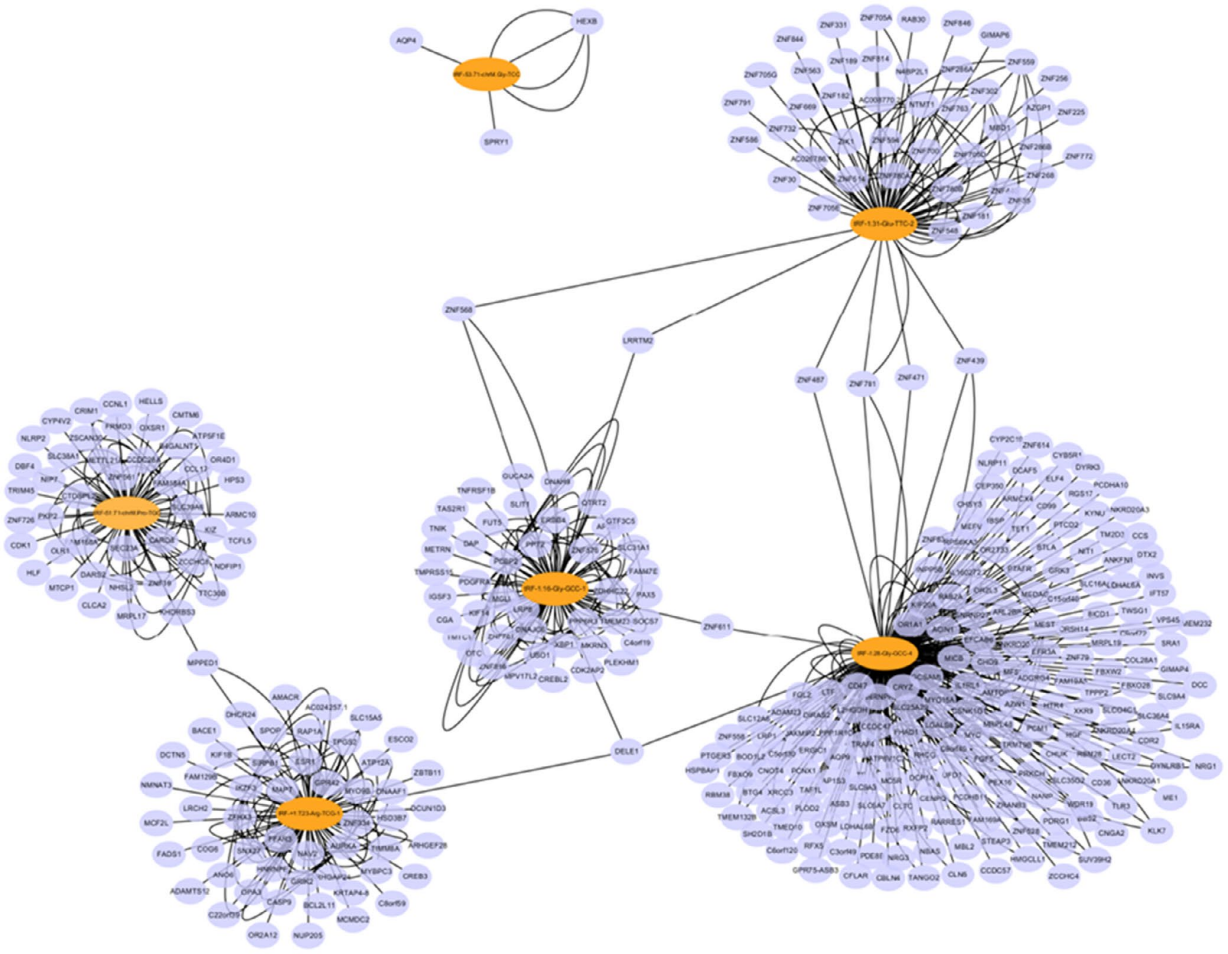
TRANSFER RNA (tRNA)‐derived fragment (tRF)‐target gene interactions. Interaction networks of tRFs and tRNA halves with target genes in atherosclerosis. The orange dot represents tRF and the purple dot represents target messenger RNAs

In order to explore the possible roles of tRFs and tiRNAs in AS, target genes for six up‐regulated and validated tRFs in the AS group were identified based on TargetScan and miRanda databases. The target genes for tRF‐1:28‐Gly‐GCC‐4, tRF‐1: tRF‐1:31‐Glu‐TTC‐2, tRF‐1:16‐Gly‐GCC‐1, tRF‐+1:T23‐Arg‐TCG‐1, tRF‐53:71‐chrM. Gly‐TCC and tRF‐51:71‐chrM. Pro‐TGG were predicted. Considering the large number of target genes, they were screened for greater binding probability according to the sum of free energy of tiRNA and target genes binding (Table S3). The network of tRF‐target gene interaction was visualized with energy <−20 in Cytoscape (Figure 5).

### 3.6 Enrichment analyses of the target genes

**FIGURE 6:**
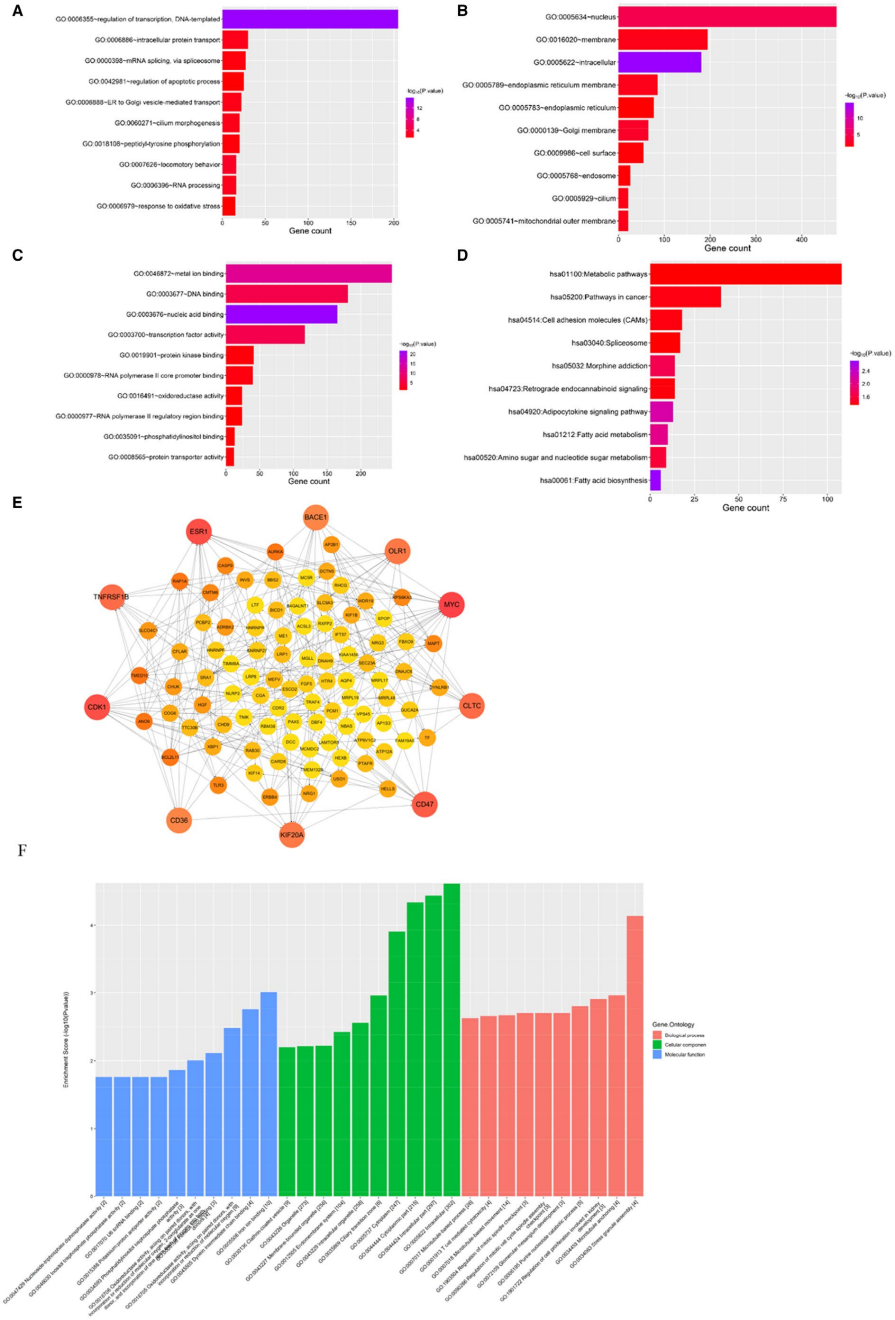
Enrichment analyses of the target genes. A, BP: biological process. B, CC: cellular component. C, MF: molecular function. D, Kyoto Encyclopedia of genes and genomes: signalling pathway. E, The protein‐protein interaction network of top 100 transfer RNA‐derived fragments. The red circles represent the 10 highest degree genes. F, Gene ontology enrichment analyses for tRF‐Gly‐GCC

In order to explore the function of target genes, analyses of GO annotation and KEGG pathway were performed using the DAVID online tool. Results of the top 10 GO and KEGG items containing the biological processes (BPs), cellular components (CCs), molecular functions (MFs) and KEGG pathways were listed in Figure 1A‐D. The most enriched items for BP included regulation of transcription DNA‐templated, intracellular protein transport and mRNA splicing via spliceosome (Figure 6A). The nucleus, membrane and intracellular were the most enriched terms for CC (Figure 6B). The most enriched terms for MF were functions in metal ion binding, DNA binding and nucleic acid binding (Figure 6C). The top 10 KEGG categories were related to metabolic and cancer pathways. Interestingly, the pathway in cell adhesion molecules (CAMs) was demonstrated to be closely associated with AS (Figure 6D). PPI network constructed to study the connections between the targets was analysed using the STRING database. As shown in Figure 6E, the top 100 linked tRFs were identified. Ten red much larger circles in the outer ring denote the top ten genes with a high‐ranking degree. They are distinguished according to the connection score from yellow to red. Interestingly, the identified target genes, including *MYC*, cyclin‐dependent kinase 1 (*CDK1*), kinesin family member 20A (*KIF20A*) and oxidized low‐density lipoprotein receptor 1 (*OLR1*), demonstrated a strong association with cell proliferation in AS. Based on FC and p‐values, tRF‐Gly‐GCC was identified for the latter experiments. The functional enrichment analysis of tRF‐Gly‐GCC was conducted to identify its potential regulatory genes (Figure 6F). Results revealed that the most enriched terms were stress granule assembly (GO:0034063) in BP, intracellular (GO:0005622) in CC and iron ion binding (GO:0005506) in MF.

### 3.7 Role of tRF‐Gly‐GCC in cell function

**FIGURE 7:**
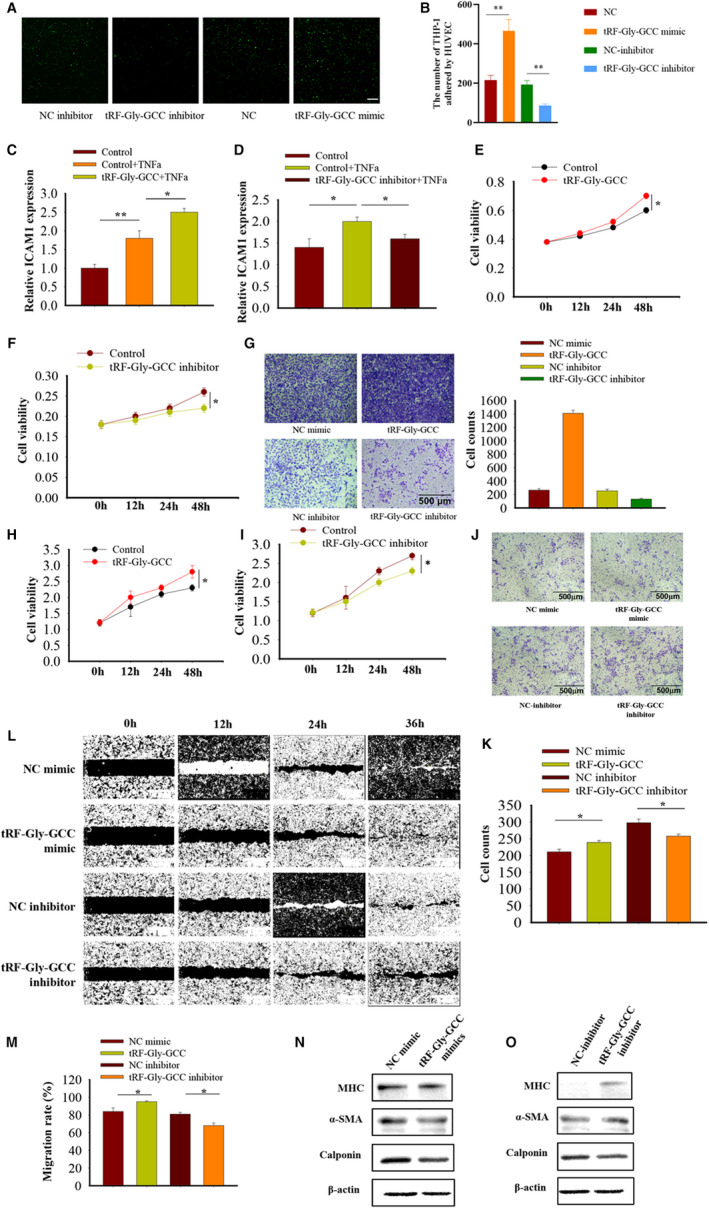
The biological roles of tRF‐Gly‐GCC in vessels cells. A, The transfection efficiency of 20 μmol/L tRF‐Gly‐GCC mimics and inhibitors in HUVECs. B, The adherent capability of THP‐1 to human umbilical vein endothelial cells (HUVECs) was measured by CFSE assay. C, The adherent cells were calculated and analysed. D and E, HUVECs were transfected with tRF‐Gly‐GCC mimics and inhibitors for 24 h, respectively, and qRT‐PCR was performed to detect adhesion molecules ICAM1 induced by TNF‐α. F and G, The ability of HUVECs proliferation was measured by Cell Counting Kit‐8 (CCK8) assay at 0, 12, 24 and 48 h. H, tRF‐Gly‐GCC effects on HUVECs migration ability assessed using transwell assay and its quantification analysis. I and J, The ability of vascular smooth muscle cells (VSMCs) proliferation was measured by CCK8 assay at 0, 12, 24 and 48 h. K and L, tRF‐Gly‐GCC effects on VSMCs invasion ability assessed using transwell assay and its quantification analysis. M, tRF‐Gly‐GCC effects on VSMCs migration ability assessed using wound healing assay and its quantification analysis. N, Representative micrographs of wound healing assay of VSMCs at 0, 12, 24 and 36 h. O and P, The protein level of VSMCs phenotype genes

Given that tRF‐Gly‐GCC was up‐regulated in atherosclerotic arterial samples, a series of gain‐of‐function experiments were performed to explore whether it plays a role in the progression of AS in HUVECs and VSMCs. Firstly, we synthesized tRF‐Gly‐GCC mimics and inhibitors, and checked their transfection efficiency (Figure 7A). We observed that there was strong overexpression and knockdown, whereas no obvious effect on tRNA‐Gly‐GCC (Figure S2). Since cell adhesion is predicted to be one of the most significant pathways among differentiated tRF/tiRNAs, cell adhesion assay was performed to confirm the same. We observed enforecd expression of tRF‐Gly‐GCC could promote adhesion of THP‐1 cells to HUVECs, whereas down‐regulation of tRF‐Gly‐GCC led to decreased adherent cell numbers (Figure 7B,C). Further, the expressions of regulatory genes *ICAM1* were also up‐regulated after transfection of tRF‐Gly‐GCC overexpression (Figure 7D) compared with suppression by tRF‐Gly‐GCC inhibitor treatment (Figure 7E). CCK8 assay revealed that overexpression of tRF‐Gly‐GCC significantly promotes HUVECs proliferation (Figure 7F), whereas knockdown of tRF‐Gly‐GCC results in attenuated proliferation (Figure 7G). The transwell assay showed that tRF‐Gly‐GCC could enhance the migration ability of HUVECs (Figure 7H). Whether tRF‐Gly‐GCC has the same effect on cell proliferation and migration in VSMCs was examined next. Consistently, transwell assay revealed that tRF‐Gly‐GCC facilitates cell proliferation (Figure 7I); knockdown resulted in attenuated cell migration (Figure 7J). The transwell assay (Figure 7K,L) and wound healing (Figure 7M,N) showed that tRF‐Gly‐GCC exerts a positive effect on cell migration in VSMCs. A close relationship between phenotype switching and proliferation and migration has been reported in VSMCs.^28^ Hence, the role of tRF‐Gly‐GCC in VSMCs phenotype switching was detected. As shown in Figure 7O,P, protein levels of contractile markers, such as MHC, were dramatically down‐regulated in tRF‐Gly‐GCC mimic‐transfected cells. On the contrary, knock down of tRF‐Gly‐GCC resulted in a clear increase in the protein level. No significant change in α‐SMA and calponin protein levels was reported. Taken together, the results indicate that tRF‐Gly‐GCC can accelerate cell proliferation and migration by negatively regulating the protein level of MHC, rather than α‐SMA and calponin.

## 4 DISCUSSION

Existing studies have shown that tRFs and tiRNAs are highly conserved small ncRNAs fragments playing important roles in physiological and pathological conditions, such as development,^29^ ageing,^30^ neurodegenerative diseases,^31^ cancer^32^, ^33^, ^34^ and cardiovascular diseases.^35^ Hence, they serve as rational therapeutic targets.^36^ In the current study, it was hypothesized that differential expression of tRFs and tiRNAs play an essential role in the development of AS. Analysis of the expression profiles of tsRNAs using RNA sequencing showed that 79 tRFs and tiRNAs were down‐regulated and 60 were up‐regulated in the AS group compared to the NC group. Furthermore, RT‐qPCR was performed to validate the six tRFs and tiRNAs that were up‐regulated in the AS group. To further ascertain the role of tRFs and tiRNAs, target genes were predicted using TargetScan and miRanda databases. In addition, GO and KEGG enrichment analyses for the targets of validated tRFs were performed. PPI network showed that 378 target genes and 10 hub genes possess a significantly close association with the tRFs using Cytoscape software. Finally, tRF‐Gly‐GCC was identified as a novel tRF that increases in AS. This increase augments proliferation and migration of HUVECs and VSMCs, indicating the need to study its potential role in the pathogenesis of AS. Results suggest that tRFs and tiRNAs might play important roles in AS.

Byproducts of tRNA cleavage, tsRNAs, are small ncRNAs of length 18‐40 nucleotides.^37^ Recently identified as rising star of the ncRNAs, tsRNAs have been proven to be associated with regulation of RNA processing and protein translation.^38^ Additional studies revealed that tRFs and tiRNAs fulfil an important function in multiple diseases. For example, high‐throughput sequencing revealed that tDR‐0009 and tDR‐7336 increased in hypoxia‐treated triple‐negative breast cancer cell lines. They could regulate hypoxia‐induced chemoresistance by activating the signal transducer and activator of transcription 3 pathway.^39^ tRF‐Leu‐CAG, which remains up‐regulated in human non‐small cell lung cancer, could bind to AURKA29 to regulate the cell cycle.^40^ Furthermore, tsRNAs have been identified as reliable biomarkers of Parkinson's disease owing to the significant differences in their expression in the prefrontal cortex and cerebrospinal fluid of patients with Parkinson’ s disease.^41^ However, expression profiles of tRFs and tiRNAs in AS tissue are yet to be reported. This study validated six tRFs using RT‐qPCR; results were consistent with the sequence data, indicating up‐regulated expression of the tRFs in AS group.

Using TargetScan and miRanda databases, 378 target genes with a standard binding energy of <−20 were identified. GO and KEGG pathway analyses revealed regulation of transcription DNA‐templated process as one of the most enriched terms of BPs. Enrichment in CAMs was confirmed to be associated with AS. Based on the results, it was found that four hub genes, including *MYC*, *CDK1*, *KIF20A* and *OLR1* had greater diagnostic potential for AS. It has been reported that mitofusin‐2 regulates proliferation of VSMCs in homocysteine‐induced AS by enhancing the binding of c‐Myc to DNA methyltransferase 1.^42^ CDK1 and KIF20A regulate cell proliferation and migration, which play a significant role in pathogenesis of AS.^43^, ^44^ *OLR1* was down‐regulated upon treatment of HUVECs with aloperine. Aloperine improved cell viability and inhibited the adhesion of monocytes to HUVECs by reducing the expression of *OLR1*.^45^ Overall, the four hub genes for six tRFs and tiRNAs might serve as potential diagnostic biomarkers in AS.

Finally, tRNA‐Gly‐GCC was identified as the novel biomarker and subjected to further experiments to unravel its role in AS. tRNA‐Gly‐GCC inhibited the expression of MERVL‐related genes that could regulate histone production.^46^ In this study, tRNA‐Gly‐GCC could promote cell adhesion, proliferation and migration of HUVECs and VSMCs by primarily managing the expression of *MHC*. However, further studies concerning tRNA‐Gly‐GCC and hub gene are warranted to explore the regulatory mechanisms of tRNA‐Gly‐GCC in the progression of AS. In addition, their functions in vivo are also to be identified.

The most important advantages of tRFs and tiRNAs that make them serve as biological diagnostic tools and therapeutic markers are their structural characteristics and chemical properties. Firstly, nucleotide modifications, such as 5‐methylcytidine and n2‐methylguanosine, increase the stability of tRFs and tiRNAs. Levels of tRFs and tiRNAs are significantly reduced in the absence of methylation, indicating that unmethylated tRFs and tiRNAs are degraded by nucleases.^47^ Stability is maintained upon binding of the tRFs and tiRNAs to serum protein complexes.^48^ Secondly, tRFs and tiRNAs are widely distributed and highly expressed in the human body. Proportion of transcriptome aligned fragments in the biological fluid is 50%. In addition, tRFs and tiRNAs are not only found in tissue cells, but also in bile, urine, seminal plasma and amniotic fluid,^49^ which reduces the difficulty in detection and facilitates extraction. In vertebrates, such as fish, amphibians, reptiles, birds, mice, non‐human primates and humans, serum tRFs and tiRNAs (at least for tsRNA‐Gly and tsRNA‐Glu) comprise highly conserved sequences, which aid in studying the effects of treatment for AS among different species in the early stage. Of note, tRFs and tiRNAs have unique expression patterns in different tissues and at different time periods. Tissue specificity and time specificity are beneficial to improve the specificity of clinical detection of tRFs and tiRNAs in the diagnosis and prognosis of atherosclerotic diseases.^47^

In summary, tRFs and tiRNAs have the potential to emerge as novel biomarkers for the prevention and treatment of atherosclerotic diseases. This study highlighted new targets and methods for the early diagnosis and treatment of patients with AS.

## CONFLICT OF INTEREST

The authors declare that they have no conflict of interest.

## AUTHOR CONTRIBUTIONS

**xiangqin he:** Data curation (lead); Formal analysis (lead); Investigation (lead); Validation (equal); Visualization (equal); Writing‐original draft (equal). **Yanyan Yang:** Conceptualization (equal); Funding acquisition (equal); Methodology (equal); Resources (equal); Software (equal); Writing‐review & editing (equal). **Qi Wang:** Formal analysis (equal); Methodology (equal); Software (equal); Validation (equal); Visualization (equal); Writing‐original draft (equal). **Jueru Wang:** Resources (supporting). **Shifang Li:** Resources (supporting). **Chunrong Li:** Resources (supporting). **Tingyu Zong:** Investigation (supporting); Methodology (supporting); Resources (supporting). **Xiaolu Li:** Investigation (supporting); Methodology (supporting); Resources (supporting). **Ying Zhang:** Investigation (supporting); Methodology (supporting); Resources (supporting). **Yulin Zou:** Investigation (supporting); Methodology (supporting); Resources (supporting). **Tao Yu:** Conceptualization (lead); Funding acquisition (lead); Project administration (lead); Supervision (equal); Validation (equal); Visualization (equal); Writing‐review & editing (lead).

## Supporting information

Supplementary Material

## Data Availability

The data of this study are available from the corresponding author upon reasonable request.
