# Supplementary material for: Expression profiles and potential roles of transfer RNA‐derived small RNAs in atherosclerosis
Source: J Cell Mol Med. 2021 Jun 16;25(14):7052–65. doi: 10.1111/jcmm.16719 (PMC8278088; doi:10.1111/jcmm.16719)

Supplementary files

Supplementary Table 1. The primers used in RT-qPCR.

tRF-1:32-Ala-AGC-4-M6	Forward: 5'-GGGGATGTAGCTCAGTGGTAGAG-3'
tRF-1:28-Gly-GCC-4	Forward: 5'-CCGCATAGGTGGTTCAGTGGTAGAA-3'
tRF-1:31-Glu-TTC-2	Forward: 5'-TATCCCACATGGTCTAGCGGTTAGG-3'
tRF-54:74-chrM.Phe-GAA	Forward: 5'-CACATCACCCCATAAACACCAA-3'
tRF-51:71-chrM.Pro-TGG	Forward: 5'-GCTAAAGACTTTTTCTCTGACCA-3'
tRF-+1:T23-Arg-TCG-1	Forward: 5'-CCAAGGGAGGTTATGATTAACTT-3'
tRF-1:16-Gly-GCC-1	Forward: 5'-GCGCATGGGTGGTTCAGTAAA-3'
tRF-1:16-Val-CAC-3	Forward: 5'-GCGTTTCCGTAGTGTAGCAAAA-3'
tRF-53:71-chrM.Gly-TCC	Forward: 5'-GCCATTCAAAAAAGAGTACCAAA-3'

Supplementary Table 2. Quality score of sequencing data.

Sample	Total Read	Total Base	Base Q30	Base Q30 (%)
AS1	13437453	685310103	626874902	91.47
AS2	7752613	395383263	359365218	90.89
AS3	7158171	365066721	334241507	91.56
AS4	7126935	363473685	330982040	91.06
Control1	4136403	210956553	191773837	90.91
Control2	9512937	485159787	440991328	90.90
Control3	7300875	372344625	339057402	91.06
Control4	6396721	326232771	298258349	91.43

Supplementary Table 3. The tRFs-target genes network.

Symbol	Up/Down	Count	Target mRNA
tRF-53:71-chrM.Gly-TCC	Up	3	HEXB, AQP4, SPRY1
tRF-51:71-chrM.Pro-TGG	Up	45	CDK1, NIP7, ZNF19, OLR1, SLC38A1, ZCCHC8, CRIM1, OXSR1, TRIM45, CTDSPL2, TTC30B, MRPL17, CMTM6, PKP2, ATP5F1E, DARS2, METTL21A, HLF, MTCP1, KIZ, NLRP2, OR4D1, CARD8, DBF4, CCDC28A, FAM168A, ZNF561, HPS3, CCL17, SEC23A, NDFIP1, ZNF726, CLCA2, B4GALNT1, TCFL5, CYP4V2, HELLS, FAM184A, KHDRBS3, ARMC10, ZSCAN30, CCNL1, SLC39A6, FRMD3, NHSL2
tRF-1:31-Glu-TTC-2	Up	43	RAB30, MBD1, NTMT1, ZNF669, ZIK1, ZNF705G, ZNF780A, ZNF705E, ZNF705A, ZNF705D, N4BP2L1, GIMAP6, ZNF331, ZNF563, ZNF732, ZNF189, ZNF559, ZNF225, ZNF35, ZNF700, ZNF440, ZNF181, ZNF286B, ZNF286A, ZNF182, ZNF586, ZNF814, AZGP1, AC026786.1, ZNF268, ZNF514, ZNF791, ZNF302, ZNF844, ZNF772, ZNF256, ZNF30, ZNF780B, ZNF846, ZNF548, ZNF594, ZNF763, AC008770.2
tRF-+1:T23-Arg-TCG-1	Up	53	BCL2L11, NAV2, C22orf39, SPOP, MYBPC3, LRCH2, AURKA, MAPT, DCTN5, ANO6, OR2A12, DHCR24, ARHGAP24, MCF2L, DCUN1D3, ZBTB11, RAP1A, SLC15A5, SNX27, ZFHX3, MYO9B, ESR1, COG6, HSD3B7, FAM129B, MPPED1, HNRNPF, ZNF334, BACE1, NUP205, C8orf59, DNAAF1, TIMM8A, CREB3, GPR42, FFAR3, ESCO2, IKZF3, KIF1B, FADS1, ARHGEF28, NMNAT3, CASP9, AMACR, MCMDC2, SIRPB1, ATP12A, TPGS2, GRIK2, ADAMTS12, KRTAP4-8, OPA3, AC024257.1
tRF-1:28-Gly-GCC-4	Up	187	EFCAB6, MRPL19, RPS6KA3, CCDC57, MFSD6, WDR19, L2HGDH, LDHAL6A, TMEM212, DCC, IL1RL1, HMGCLL1, SLC36A4, XRCC3, NRG3, CNOT4, CHD9, CD36, ELF4, OR1A1, MICB, KLK7, SLC5A7, UFD1, PTGER3, ADAM23, GCSAML, SLC9A4, SLC25A29, BTG4, MBL2, RXFP2, ASB3,

			GPR75-ASB3, PCNX1, C6orf120, TRMT9B, SH2D1B, PDRG1, MYO15A, CHSY3, ME1, HTR4, ZNF614, SLC12A6, OXSM, AL160272.2, BICD1, DYRK3, BOD1L2, ADGRG4, STEAP3, FBXO9, ZRANB3, ZNF439, AQP9, C3orf49, TMEM132B, CENPQ, DIRAS2, TMEM232, FBXW2, CDR2, C5orf30, PCDHA10, NANP, RFX5, LRP1, LECT2, AP1S3, PTCD2, INVS, NLRP11, SLC9A3, CD99, KIF20A, LDHAL6B, KYNU, DCAF5, BBS2, TM2D3, MEST, CEP350, CBLN4, AZIN1, HSPBAP1, ANKFN1, ARMCX4, ZCCHC4, VPS45, RBM28, COL28A1, XKR9, GIMAP4, CRYZ, CYB5R1, FHAD1, MRPL48, CHUK, CLN5, ERGIC1, PEX16, PCDHB11, TET1, CSNK1G1, C9orf72, TAF1L, RAB2A, BTLA, HGF, ZNF487, FGL2, SLC35G2, LTF, DTX2, SLC16A14, DCP1A, PPP1R1C, LAMTOR5, JAKMIP2, EFR3A, SLCO4C1, CD47, MC5R, ACSL3, TLR3, RARRES1, SUV39H2, IFT57, INPP5B, FZD6, NBAS, FBXO28, ARL2BP, RBM38, NRG1, ZNF830, TWSG1, OR2T33, ANKRD20A1, ANKRD20A3, ANKRD20A2, ANKRD20A4, IBSP, TRAF4, PTAFR, CCS, TF, HNRNPR, MEDAG, FAM169A, LGALS8, ATP6V1C2, C15orf40, OR2L3, CCDC47, CNGA2, ZNF558, GRK3, ZNF528, SNRNP27, PLOD2, MEFV, NIT1, IL15RA, CLTC, TPPP2, ZNF471, PDE8B, CYP2C19, ACIN1, SRA1, RHCG, CFLAR, C9orf40, PRKCH, TANGO2, TMED10, ZNF79, FAM19A5, PCM1, FGF5, OR5H14, RGS17, MYC, ZNF781, DYNLRB1
tRF-1:16-Gly-GCC-1	Up	47	OTC, CREBL2, PCBP2, TAS2R1, DNAJC6, DNAH9, CGA, TMTC1, LRP8, PPP6R3, USO1, FAM47E, IGSF3, XBP1, PDGFRA, QTRT2, ZNF576, MGLL, DELE1, FUT5, SLC31A1, CDK2AP2, MPV17L2, ZNF761, ZNF816, KIF14, C4orf19, DAP, SOCS7, METRN, ZNF611, PPT2, TNIK, GTF3C5, TMEM234, LRRTM2, ZNF568, ERBB4, PLEKHM1, GUCA2A, PAX5, MKRN3, SLIT1, TNFRSF1B, AP2B1, TMPRSS15, ZDHHC22

Supplementary figure 1 The expression level of tRNA-Gly-GCC in clinical samples.

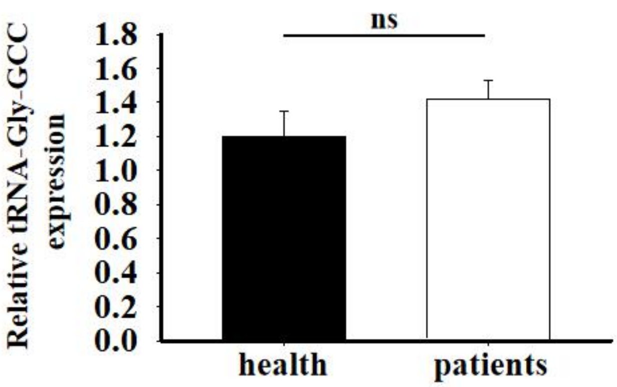

Supplementary figure 2 The expression level of tRNA-Gly-GCC after transfection of tRF-Gly-GCC in VSMCs.

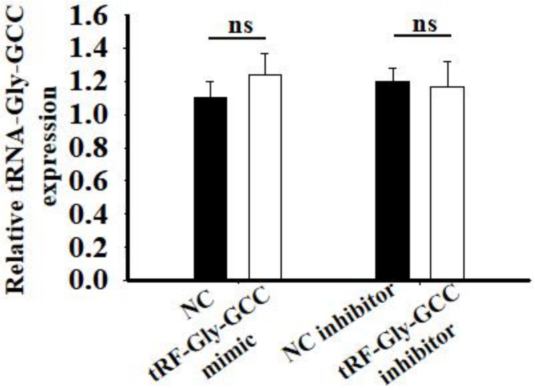

Supplement: Supplementary file 1 — Supplementary Material [file JCMM-25-7052-s001.pdf]
